# Mechanism of the action of bioactive proteins of vegetables in diabetes mellitus type 2: Systematic review protocol

**DOI:** 10.1097/MD.0000000000017326

**Published:** 2019-09-27

**Authors:** Amanda Fernandes de Medeiros, Izael de Sousa Costa, Grasiela Piuvezam, Gidyenne Christine Bandeira Silva de Medeiros, Bruna Leal Lima Maciel, Ana Heloneida de Araújo Morais

**Affiliations:** a Biochemistry Postgraduate Program, Biosciences Center; b Collective Health Postgraduate Program (PPGSCoL), Center for Health Sciences; c Nutrition Postgraduate Program, Center for Health Sciences, Federal University of Rio Grande do Norte, Natal, RN, Brazil.

**Keywords:** antidiabetic peptide, diabetes mellitus, hypoglycemic agents, systematic review, type 2, vegetable proteins

## Abstract

**Background:**

Diabetes mellitus type 2 (DM2) is a chronic disease of significant prevalence causing hyperglycemia and several comorbidities. Evidences highlight the performance of non - protein bioactive compounds found in vegetables in the control of hyperglycemia. This study describes a protocol of a systematic review, which analyzes the action of proteins and bioactive peptides of plants in DM2.

**Methods:**

The Preferred Reporting Items guide this protocol for Systematic Reviews and Meta-Analyzes Protocols (PRISMA-P) was used. The databases that will be used for searching will be PubMed, ScienceDirect, Scopus, Web of Science, EMBASE, and Virtual Health Library, Brazil (VHL). Studies that use bioactive proteins and peptides of vegetal origin in DM2 will be included in the systematic review. The studies will be identified using clinical parameters and the effect on insulin resistance. The characteristics of the studies as control groups, test substance, dosage, intervention time, and the main results will be described. Selection of studies, data extraction, and methodological quality assessment will be performed independently by two experienced reviewers.

**Results:**

This protocol will be the basis for a systematic review identifying the mechanism of action of plant proteins and peptides in type 2 diabetes mellitus.

**Conclusion:**

Systematic reviews from this protocol will provide support for the construction of researches that analyze the effect of plant bioactive proteins and peptides on the control of hyperglycemia and how these molecules act in the control of DM2.

**Prospero Registration Number:**

CRD42019110956.

## 1 Introduction

Diabetes mellitus (DM) is a metabolic disease characterized by the state of hyperglycemia. This condition results from changes in insulin secretion and action. Besides, the impairment of insulin secretion and the low sensitivity to the action of this hormone often coexist in the same patient. It is not clear at what point in the metabolic pathway there is an abnormality that triggers the development of the hyperglycemic state.^[1]^

DM is classified as type 1, type 2, gestational, and others. Diabetes mellitus type 2 (DM2) affects between 90% and 95% of people with diabetes, including individuals with insulin resistance and with a relative deficiency of insulin.^[1]^ The risk of developing this type of diabetes increases with age; the presence of comorbidities, such as obesity; diets rich in simple carbohydrates and lack of physical activity.^[2]^ The standard treatment of DM2 is aimed at changes in lifestyle to promote glycemic control, with the practice of physical exercise and healthy eating habits; the use of pharmacotherapy is indicated in some situations.^[3]^ The literature indicates that in DM2, several metabolic complications occur in the micro and macrovascular environments, such as retinopathy, nephropathy and cardiovascular diseases, such as stroke, respectively.^[4]^ Concomitant, changes in the inflammatory profile, increased oxidative stress, and altered protein metabolism are well documented.^[4–6]^

Due to the high prevalence and complications associated with the disease, alternative treatments for DM2 are sought to bring the quality of life to the individuals affected by this disease. Lo and Wasser,^[7]^ in their review, verified the applicability of bioactive compounds of plant origin with the potential to minimize and control DM2 and its associated metabolic alterations. In that review, the action of bioactive compounds on DM2 was observed, with emphasis on the polyphenols^[8,9]^ and polysaccharides.^[10,11]^

It is also worth mentioning the studies developed with proteins or peptides of plant origin that show the effect of these molecules on obesity and metabolic syndrome.

An example is a bioactive protein extracted from a tropical fruit called Tamarindo Isolated Trypsin Inhibitor (TTI).^[12]^ Ribeiro et al^[13]^ observed that TTI presented a satietogenic effect, reducing, in eutrophic rats, the weight gain associated with the serum increase of cholecystokinin (CCK). The studies by Carvalho et al^[14,15]^ found that TTI in addition to reducing food intake in animals with obesity and metabolic syndrome also affected the reduction of TNF-α and a slight reduction in the mean concentrations of glycemia. This last finding is important for the development of studies to verify the action of bioactive plant proteins on DM2.

In this perspective, when it comes to understanding the mechanisms of action prior to clinical application of bioactive proteins or peptides of plant origin, most studies are developed in animal models. These experiments are important for studying the development of new medical therapies of interventions for humans. In this sense, awareness has increased about the importance of conducting systematic reviews in the field of laboratory animal experimentation.^[16]^ Nowadays, it's common systematic reviews and systematic reviews protocols on animal therapies and interventions.^[17,18]^

Thus, knowing that proteins of vegetable origin act in the most diverse clinical conditions.^[19]^ Considering the progression of studies aimed at the performance of bioactive proteins and their implications in the diverse conditions of the individual. It is necessary to develop a systematic review protocol to analyze the action of isolated or purified bioactive proteins of vegetables on the control of hyperglycemia and DM2.

## 2 Methods

### 2.1 Protocol and registration

**Figure 1:**
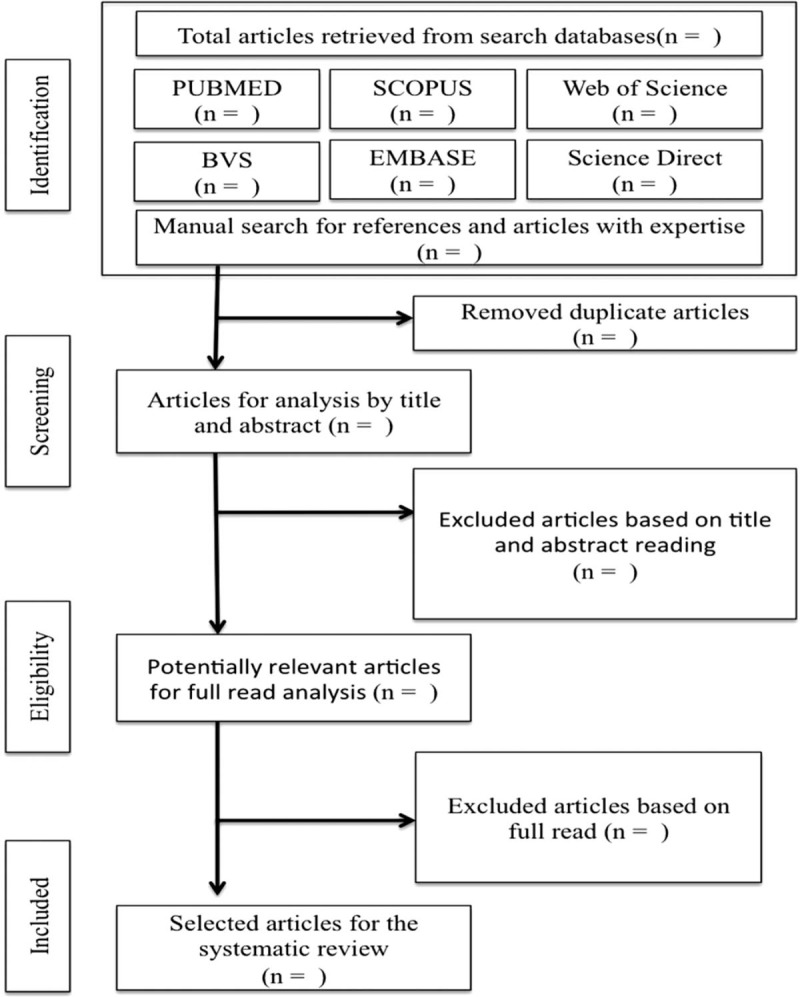
Flowchart for selection of study articles based on PRISMA-P.

This systematic review (SR) protocol was registered in the International Prospective Register of Systematic Reviews (PROSPERO) database, on the 19th of June 2019, under the protocol number: CRD42019110956, and is available at: https://www.crd.york.ac.uk/prospero/display_record.php?ID=CRD42019110956. The methodology for constructing the study will follow the criteria established by the Preferred Reporting Items for Systematic Reviews and Meta-Analyzes Protocols (PRISMA-P) (Fig. 1).^[20]^ This study will be based on scientific literature, so approval by ethics committees will not be necessary.

### 2.2 Eligibility criteria

As the criteria of eligibility, the articles should meet the PICO criteria (Problem, Intervention, Control, and Outcomes), described below.

#### 2.2.1 Inclusion criteria

##### 2.2.1.1 Participants

Male mouse/mice with experimental type 2 diabetes mellitus (all species) will be used, with or without obesity.

##### 2.2.1.2 Types of intervention

Treatment of diabetes with purified proteins or peptides of vegetable origin. All timings, frequencies and dosages of treatment are eligible for inclusion.

##### 2.2.1.3 Types of controls

Control animals treated with vehicle and/or with known drugs for diabetes mellitus (e.g., insulin, metformin) for inclusion in the systematic review.

##### 2.2.1.4 Outcomes measure

Reduced insulin resistance and/or reduced blood glucose levels by measuring blood glucose and/or insulin dosage will be included.

#### 2.2.2 Exclusion criteria

##### 2.2.2.1 Participants

Other animals, animals with experimental diabetes with co-morbidities (except obesity), ex vivo, in vitro or in silico models.

##### 2.2.2.2 Types of intervention

Treatment of diabetes with vegetal extract, non-protein vegetable origin and protein of animal origin.

##### 2.2.2.3 Types of controls

No group control, control without specifications or studies without a separate control group

##### 2.2.2.4 Outcomes measure

Outcomes without reduction insulin resistance and/or reduced blood glucose levels

### 2.3 Information sources and literature search

To identify the studies that will be included in the review, it is necessary to develop search strategies from keywords indexed in the Medical Subject Headings (MeSH). The descriptors used will be “diabetes mellitus 2”, as studied disease, the dietary intervention, that will be “isolated” or “purified bioactive proteins” or “peptides of plant origin”; and the expected results will be the “reduction of glycemia” and “improvement in insulin sensitivity”. These descriptors will be accompanied by the Boolean operators OR and AND (Table 1).

**Table 1:**
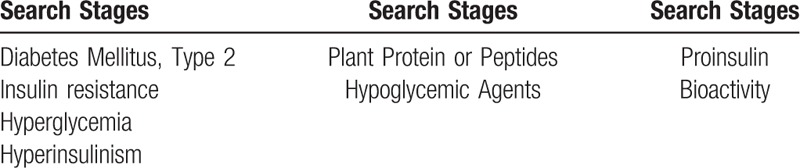
Search strategies for the analysis of the articles that will make up the systematic review.

The search strategies will be applied in the following electronic databases: PubMed, ScienceDirect, Scopus, Web of Science, EMBASE, and Virtual Health Library, Brazil (VHL). The searches in these databases will be carried out on a computer with the IP of the Federal University of Rio Grande do Norte (UFRN), which gives open access to all articles of each indexed database, through the “Portal de Periódicos Capes” in Brazil. The EMBASE survey will be conducted through the University of São Paulo (USP).

Bibliographic research will be carried out concomitantly by 2 researchers, and the initial searches will test preliminary equations with the perspective of applying search strategies with high specificity.

At the end of the database searches, the articles will be compiled in the bibliographic reference manager Mendeley and duplicate articles will be counted and removed. Subsequently, the titles and abstracts will be read by 2 researchers who will select the articles according to the eligibility criteria. Afterward, the complete article and its references will be analyzed, and the appropriate studies will be selected for the SR inclusion criteria (Table 2).

**Table 2:**
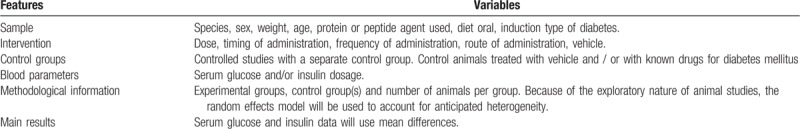
Characteristics and methodological aspects of selected studies.

### 2.4 Data extraction

Two researchers will be responsible for analyzing the data, which will be inserted into a table in Microsoft Word software. Doubts will be clarified with the help of a third researcher. Information on the characteristics, methodological aspects, and main results of the selected studies will be collected as described in Table 2.

### 2.5 Risk of bias and/or quality assessment

Two evaluators will perform the readings independently. Discrepancies will be solved with the help of a third researcher. The reviewers will have been previously trained and calibrated to ensure uniformity in the evaluation of the criteria, and Cohen kappa concordance coefficient will be applied. The evaluation of the evidence and the strength of the recommendations of the studies will be evaluated with the tool SYstematic Review Center for Laboratory animal Experimentation - SYRCLE.^[21]^

The SYRCLE, which is one of the Risk of Bias (Rob) tools, consists of ten questions that can be used to select and detect the performance, friction and biases of the scientific articles to be included in the studies. SYRCLE items will be scored “yes” indicating low risk of bias; No indicates a high risk of bias; or “not clear” indicating that the item was not reported and therefore the risk of bias was unknown.

SYRCLE tool item 1 evaluates sequence generation, and will be scored “yes” when the authors clearly describe a random component in sequence generation, such as the use of a random number manager. It is scored “no” when a nonrandom approach is applied, such as allocation by judgment or preference. Item 2 of the SYRCLE tool, group pairing, will be scored “yes” when the intervention and control group were matched for age, gender and lineage. Item 3 focuses on allocation concealment and is scored based on the method applied to conceal the allocation sequence; Suitable methods will be sequential numbering and sealed envelopes. Item 4, random allocation, will be scored “yes” when animals are randomly allocated to the experiment. Caregiver blinding, item 5, focuses on caregiver blindness to knowledge of what intervention each animal received. Appropriate blindness includes identical housing for exposure and control groups. Item 6 will be “yes” when the results evaluation is performed randomly for all animals without group distinction. Item 7 will be “yes” when the results evaluator is blinded. Item 8, incomplete results data, will be “yes” when all animals in the sample are included in the analyses and if the reasons for exclusion are clearly addressed and explained. Item 9, selective reporting of results, will be scored “yes” when all results mentioned in the methods section are reported in the results section and vice versa and if biased reporting is present. And in item 10, other potential sources of bias will be scored “yes” when other issues are identified that could result in a high risk of bias.

### 2.6 Data analysis and synthesis

The systematic review will describe the relevant information of included studies, such as experimental groups, control group(s) and the number of animals per group, species, sex, weight, age, protein or peptide agent used, oral diet, induction type of diabetes. Besides, the dose, timing of administration, frequency of administration, route of administration, and the vehicle will be observed. The results of the selected articles will show impacts on: serum glucose and/or insulin (mg / dL) in addition to the Index HOMA-IR, Glycated hemoglobin (HbA1C), Oral Glucose Tolerance Test (OGTT), Oral Insulin Tolerance Test (OITT) in the original articles.

## 3 Discussion

The systematic review proposal presented in this protocol aims to identify studies that present the mechanism of action of bioactive proteins and peptides of plant origin on the reduction of glycemia in experimental models of type 2 diabetes mellitus.

The literature has revisions of scientific literature that address the theme of DM2 relating to possible therapeutic agents of plant origin. In this sense, we highlight the study of medicinal mushrooms containing compounds with medicinal potential: polysaccharides, proteins, fibers, and antioxidant components. In this review, the types of mushrooms that may be promising was discussed.^[22]^

Another review study discussed the action of polyphenols of plant origin that include, among others, phenolic acids, flavonoids, stilbenes, lignans, and polymer lignans. From the in vivo and in vitro experiments, this review mentions that vegetable polyphenols and products rich in polyphenols act by modulating the metabolism of carbohydrates and lipids, helping to reduce hyperglycemia, dyslipidemia and insulin resistance, improving the metabolism of adipose tissue and attenuating oxidative stress, in addition to stress-sensitive signaling pathways and inflammatory processes. Besides, due to the small number of studies carried out in humans, they propose the development of clinical studies to identify the ideal and safe dose, as well as the duration of supplementation with polyphenolic compounds in diabetic patients.^[23]^

In another review study published more recently, the performance of hydrolyzed peptides derived from soybean and egg was observed. The study addressed the hydrolyzed peptides of plant and animal origin to assist in the control of glucose homeostasis; however, molecular targets were not identified in this publication.^[19]^

Experimental studies with primary data work with extracts from several plants, but it has not been possible to state which component or which components interact to cause an improvement in the DM2 profile yet. In this sense, the studies with Euterpe oleracea Mart (Amai),^[24]^ Gelidium elegans,^[25]^ Litchi chinensis Seeds,^[8]^ Codonopsis lanceolata,^[26]^ Panax notoginseng^[27]^ stand out.

These studies show a gap in the literature regarding the mechanism of action of proteins and peptides, whether isolated or purified from plant origin on DM2. Thus, considering that DM2 is a metabolic disease with high prevalence, it is necessary to develop reviews that identify the potential of these studies, as well as to elucidate the mechanisms of action to control hyperglycemia. The present protocol aims to guide the study of the effect of plant proteins on DM2 in experimental models, through a list of studies identified in the scientific literature, in which it will seek to compile the principal results and thus direct current and future researchers of this thematic area.

## Author contributions

**Conceptualization:** Amanda Fernandes de Medeiros, Izael de Sousa Costa, Grasiela Piuvezam, Ana Heloneida de Araújo morais.

**Data curation:** Amanda Fernandes de Medeiros, Izael de Sousa Costa, Gidyenne Christine Bandeira Silva de Medeiros.

**Formal analysis:** Amanda Fernandes de Medeiros, Izael de Sousa Costa, Grasiela Piuvezam, Gidyenne Christine Bandeira Silva de Medeiros.

**Funding acquisition:** Ana Heloneida de Araújo morais.

**Investigation:** Amanda Fernandes de Medeiros, Izael de Sousa Costa, Grasiela Piuvezam, Gidyenne Christine Bandeira Silva de Medeiros.

**Methodology:** Amanda Fernandes de Medeiros, Izael de Sousa Costa, Grasiela Piuvezam, Gidyenne Christine Bandeira Silva de Medeiros.

**Project administration:** Grasiela Piuvezam, Ana Heloneida de Araújo Morais.

**Supervision:** Grasiela Piuvezam, Gidyenne Christine Bandeira Silva de Medeiros, Ana Heloneida de Araújo morais.

**Writing – original draft:** Amanda Fernandes de Medeiros, Izael de Sousa Costa.

**Writing – review & editing:** Amanda Fernandes de Medeiros, Izael de Sousa Costa, Grasiela Piuvezam, Gidyenne Christine Bandeira Silva de Medeiros, Bruna Leal Lima Maciel, Ana Heloneida de Araújo morais.

Ana Heloneida de Araújo morais orcid: 0000-0002-6460-911X.
